# Supplementary material for: Notch signalling mediates reproductive constraint in the adult worker honeybee
Source: Nat Commun. 2016 Aug 3;7:12427. doi: 10.1038/ncomms12427 (PMC4976197; doi:10.1038/ncomms12427)
Supplement: Supplementary Information — Supplementary Figures 1 - 8, Supplementary Tables 1 and 2, Supplementary Notes and Supplementary References [file ncomms12427-s1.pdf]

Supplementary Figures

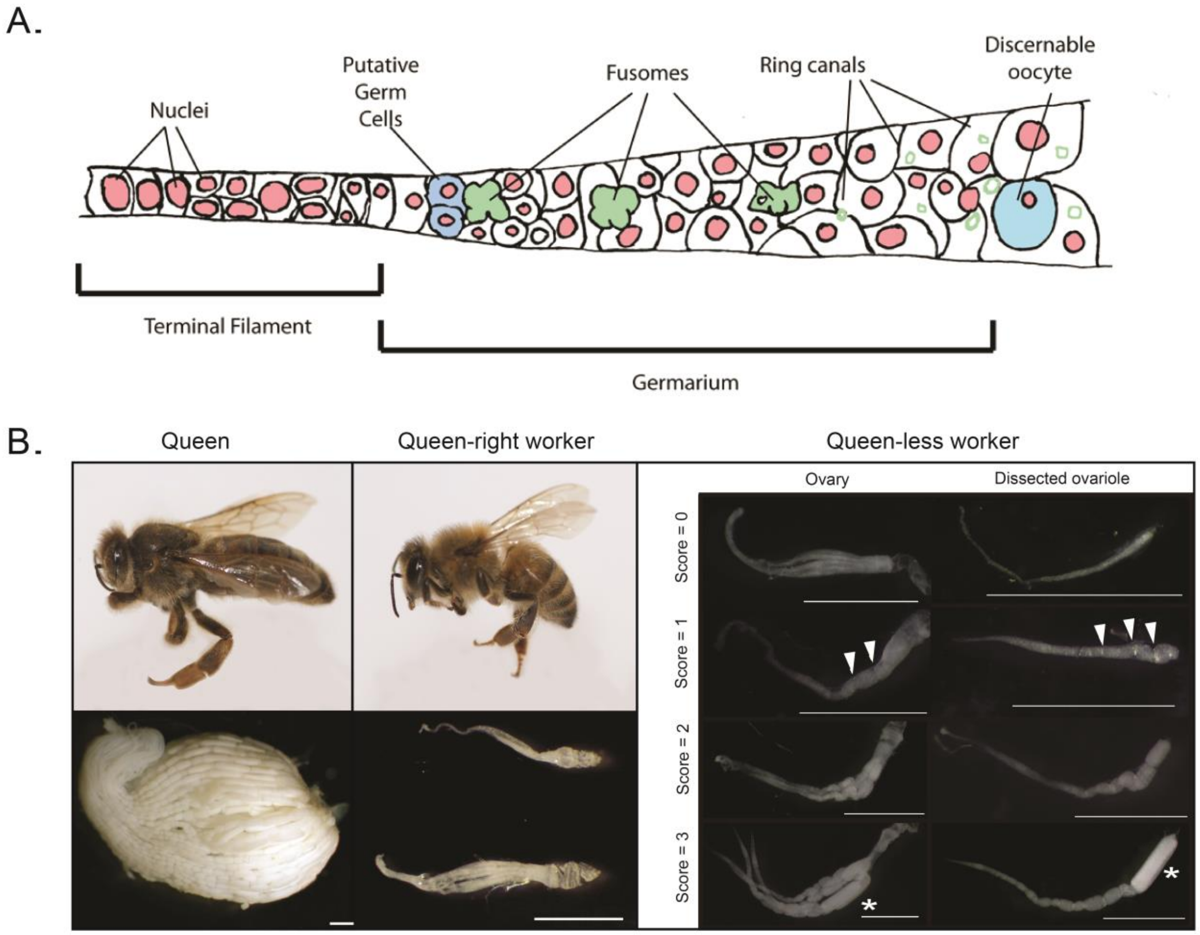

Supplementary Figure 1. Organization and morphology of honeybee ovaries.
(**A**) Diagram illustrating general features of cell organization in the terminal filament and germarium of the honeybee ovariole. Putative germ-line stem cells, housed at the anterior of the ovariole, undergo asymmetrical mitotic divisions with incomplete cytokinesis to generate cystocyte clusters that are associated with the actin rich polyfusome structures. Towards the posterior of the germarium one cell of the cystocyte cluster enlarges, this cell is destined to become the oocyte and the remaining cells of the cystocyte cluster differentiate into polyploid nurse cells. The polyfusome gives rise to a complex ring canal structure that link the cells of the cystocyte cluster (destined to become nurse cells) with the oocyte for the remainder of oogenesis.

Previous research has observed pycnotic nucleic in the germarium in worker bees and it is thought that the control of reproduction in worker bees occurs in this region of the ovariole[1]. (**B**) Queen bees have large active ovaries that consist of up to 200 ovarioles. In contrast, queen-right worker bees have small-undeveloped ovaries with 2-10 ovarioles, these ovarioles are thin and generally show no signs of cell differentiation. In the absence of a queen and her brood a proportion of worker bees undergo ovary activation. This process can be separated into four discrete stages or scores based on a modified Hess scale[2] as detailed in the text. Arrowheads indicate signs of cell differentiation and asterisks indicate mature oocytes. Scale bars indicate 1 mm.

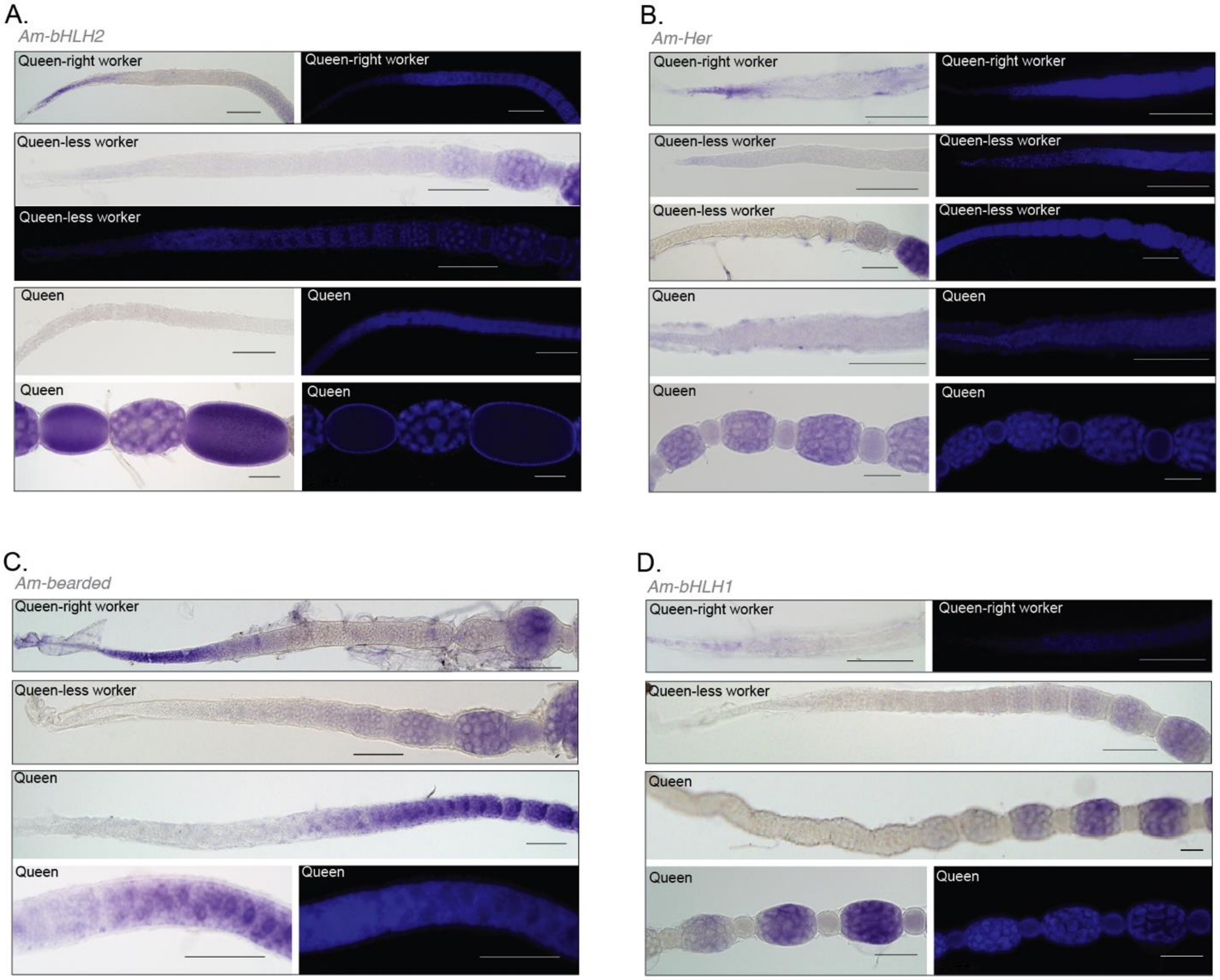

Supplementary Figure 2. Expression of genes of the E(spl)-C.

(**A**) *bHLH2* RNA is detected in the anterior germarium of queen-right worker bees, but not in the germarium of queen-less or queen bees. In queen-less worker and queen ovaries expression of *bHLH2* is restricted to the nurse cells and RNA is detected in the developing oocyte. RNA for *bHLH2* is also detected in the follicle cells overlying the developing oocyte. (**B**) *Her* RNA is detected in the anterior germarium of queen-right worker bees, but not in the germarium of queen-less or queen bees. In queen-less worker and queen ovaries expression of *bHLH2* is restricted to the nurse cells and RNA is detected in the developing oocyte. (**C**) *Bearded* RNA is detected in the anterior germarium of queen-right worker ovaries, but not in the anterior germarium of queen-less worker or queen bees. *Bearded* RNA is, however, detected in the posterior germarium of queen bees with RNA detected in the presumptive oocytes as they

enlarge and differentiate within the cystocyte cluster. (**D**) *bHLH2* RNA is weakly detected in the germarium of queen-right worker bees and in the nurse cells of queen-less worker bees and queens. *bHLH2* RNA is only weakly detected in the developing oocytes. Following *in situ* hybridisation ovaries were counter-stained with DAPI (right panels). Scale bars indicate 100 μm.

A.

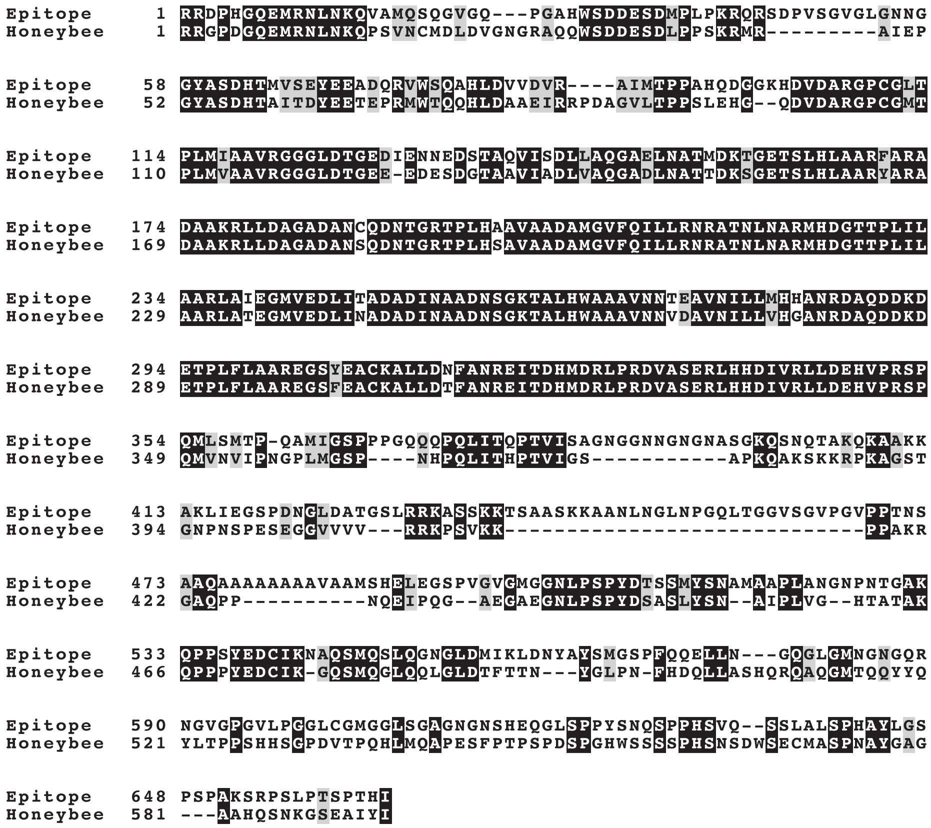

Epitope 1 RRDPHGQEMRNLNKQVAMQSQGVGQ---PGAHWSDDESDMPLPKRQRSDPVSGVGLGNNG
Honeybee 1 RRGPDGQEMRNLNKQPSVNCMDLDVGNGRAQQWSDDESDLPPSKRMR---------AIEP
Epitope 58 GYASDHTMVSEYEEADQRVWSQAHLDVVDVR----AIMTPPAHQDGGKHDVDARGPCGLT
Honeybee 52 GYASDHTAITDYEETEPRMWTQQHLDAAEIRRPDAGVLTPPSLEHG--QDVDARGPCGMT
Epitope 114 PLMIAAVRGGGLDTGEDIENNEDSTAQVISDLLAQGAELNATMDKTGETSLHLAARFARA
Honeybee 110 PLMVAAVRGGGLDTGEE-EDESDGTAAVIADLVAQGADLNATTDKSGETSLHLAARYARA
Epitope 174 DAAKRLLDAGADANCQDNTGRTPLHAAVAADAMGVFQILLRNRATNLNARMHDGTTPLIL
Honeybee 169 DAAKRLLDAGADANSQDNTGRTPLHSAVAADAMGVFQILLRNRATNLNARMHDGTTPLIL
Epitope 234 AARLAIEGMVEDLITADADINAADNSGKTALHWAAAVNNTEAVNILLMHHANRDAQDDKD
Honeybee 229 AARLATEGMVEDLINADADINAADNSGKTALHWAAAVNNVDAVNILLVHGANRDAQDDKD
Epitope 294 ETPLFLAAREGSYEACKALLDNFANREITDHMDRLPRDVASERLHHDIVRLLDEHVPRSP
Honeybee 289 ETPLFLAAREGSFEACKALLDTFANREITDHMDRLPRDVASERLHHDIVRLLDEHVPRSP
Epitope 354 QMLSMTP-QAMIGSPPPGQQQPQLITQPTVISAGNGGNNGNGNASGKQSNQTAKQKAAKK
Honeybee 349 QMVNVIPNGPLMGSP----NHPQLITHPTVIGS-----------APKQAKSKKRPKAGST
Epitope 413 AKLIEGSPDNGLDATGSLRRKASSKKTSAASKKAANLNGLNPGQLTGGVSGVPGVPPTNS
Honeybee 394 GNPNSPESEGGVVVV---RRKPSVKK-----------------------------PPAKR
Epitope 473 AAQAAAAAAAAVAAMSHELEGSPVGVGMGGNLPSPYDTSSMYSNAMAAPLANGNPNTGAK
Honeybee 422 GAQPP----------NQEIPQG--AEGAEGNLPSPYDSASLYSN--AIPLVG--HTATAK
Epitope 533 QPPSYEDCIKNAQSMQSLQGNGLDMIKLDNYAYSMGSPFQQELLN---GQGLGMNGNGQR
Honeybee 466 QPPPYEDCIK-GQSMQGLQQLGLDTFTTN---YGLPN-FHDQLLASHQRQAQGMTQQYYQ
Epitope 590 NGVGPGVLPGGLCGMGGLSGAGNGNSHEQGLSPPYSNQSPPHSVQ--SSLALSPHAYLGS
Honeybee 521 YLTPPSHHSGPDVTPQHLMQAPESFPTPSPDSPGHWSSSSPHSNSDWSECMASPNAYGAG
Epitope 648 PSPAKSRPSLPTSPTHI
Honeybee 581 ---AAHQSNKGSEAIYI

B.

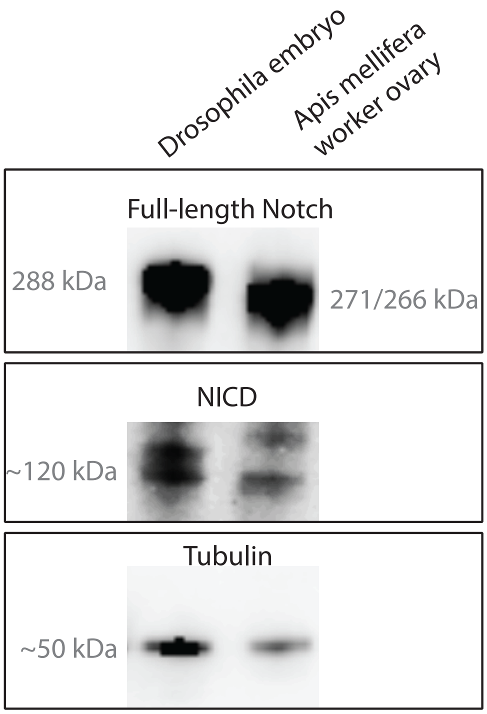

Drosophila embryo
Apis mellifera worker ovary
Full-length Notch
288 kDa
271/266 kDa
NICD
~120 kDa
Tubulin
~50 kDa

Supplementary Figure 3. Cross reactivity of the *Drosophila* Notch antibody (C17.9C6). (**A**) Sequence conservation between *Drosophila* and *Apis mellifera* for the fragment of Notch protein used to generate the C17.9C6 antibody[3]. (**B**) The C17.9C6 antibody detects proteins of the expected sizes in the *Drosophila* lysate and honeybee lysates. The most abundant immunoreactive species is consistent in size with the full-length Notch receptor (288 kDa in *Drosophila*, and 266-71 kDa in honeybee). The band that we detect in the honeybee ovary lysate is smaller in size than that seen in *Drosophila*, as consistent with bioinformatic predictions of the size of the full-length Notch receptor. We also detect two species of around 120 kDa in *Drosophila* that are consistent in size with the cleaved Notch receptor. Two similar sized species are detected in the honeybee lysate. Blots were stripped and incubated with the tubulin antibody (E7) to allow comparisons of protein loading between lanes.

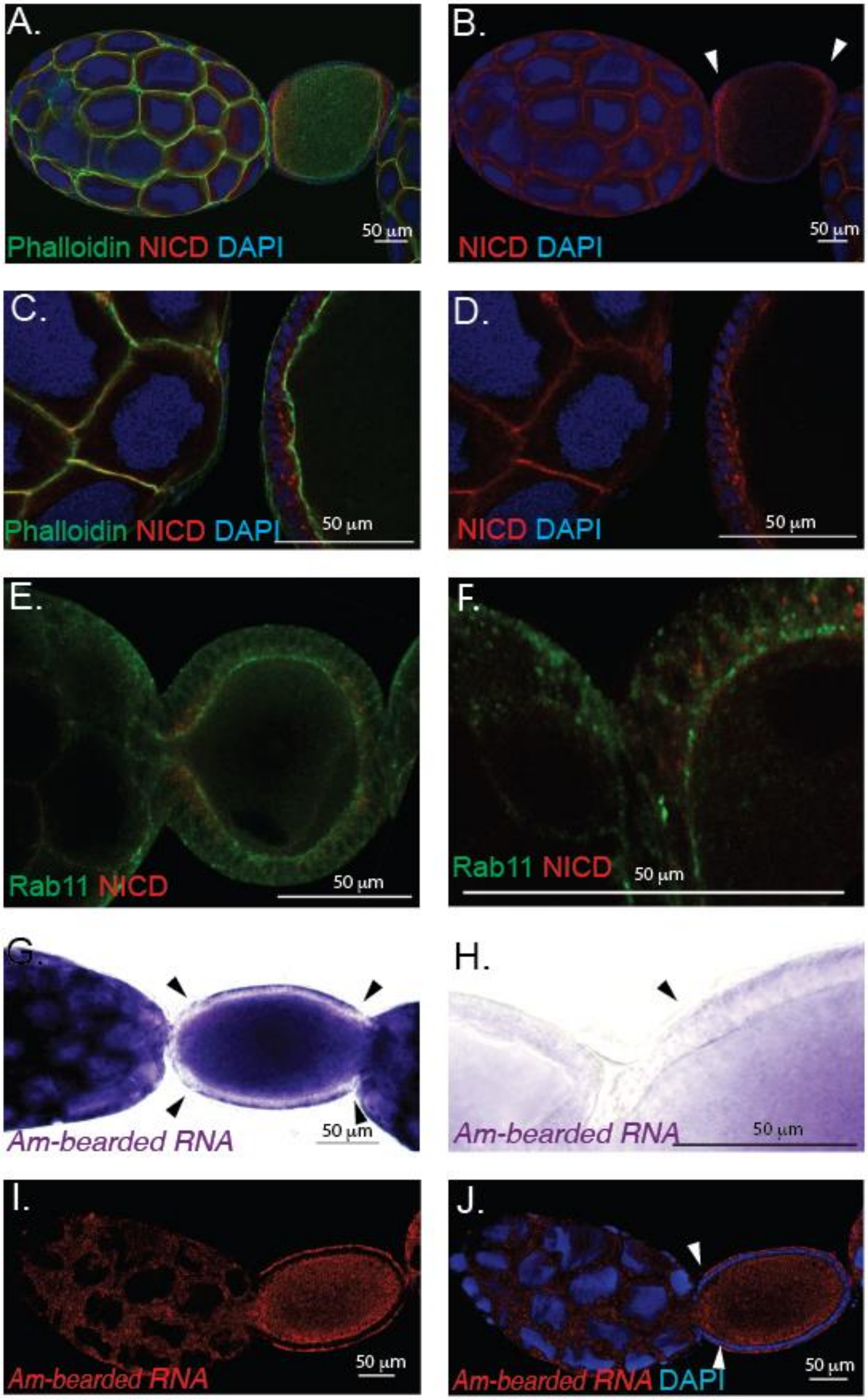
A.
Phalloidin NICD DAPI
50 µm
B.
NICD DAPI
50 µm
C.
Phalloidin NICD DAPI
50 µm
D.
NICD DAPI
50 µm
E.
Rab11 NICD
50 µm
F.
Rab11 NICD
50 µm
G.
Am-bearded RNA
50 µm
H.
Am-bearded RNA
50 µm
I.
Am-bearded RNA
50 µm
J.
Am-bearded RNA DAPI
50 µm

Supplementary Figure 4. Notch signalling differentiates follicle cell populations in the queen ovariole.

The Notch receptor is detectable on nurse cell membranes and follicle cell membranes (**A**). However, as early as stage 3 of oogenesis we see increased fluorescence associated with the follicle cells located at the anterior and posterior of the egg chamber (**B**, indicated by arrowheads). Closer examination of these anterior and posterior follicle cells indicates that the Notch receptor is present in punctate dots within the cytoplasm of these cells as well as the cell membrane (**C**). These Notch receptor-rich dots do not colocalize with nuclei (**D**), nor do they colocalize with Rab11, a marker for recycling endosomes (**E**, and higher magnification in **F**). This raises the possibility that the Notch receptor is sequestered in these anterior and posterior follicle cells rendering them refractory to Notch signalling. Consistent with this hypothesis, *in situ* hybridisation for *bearded,* a gene known to be responsive to Notch signalling, detects RNA in the main body follicle cells but not the anterior and posterior follicle cells (arrowheads in **G**). This is most obvious at the anterior of the oocyte where *bearded* RNA is not detected in the five to ten follicle cells either side of the connection with the attached nurse cells (**H**). Examining these specimens by confocal reflectance microscopy confirms that the anterior follicle cells that are in the same plane as the germinal lumen do not express *bearded* (**I**) RNA for bearded is enriched at the anterior periphery of the follicle cells proximal to the nucleus (**J**). This indicates that the main body follicle cells are receiving a Notch signal, possibly via Delta signalling from the germline. In contrast, the cells at the anterior and posterior of the egg chamber to not express *bearded*, consistent with these cells being refractory to Notch signalling from the germline. Following immunohistochemistry ovaries were counter-stained with DAPI and phalloidin to visualize nuclei and cortical-actin. Scale bars indicate 50 µm.

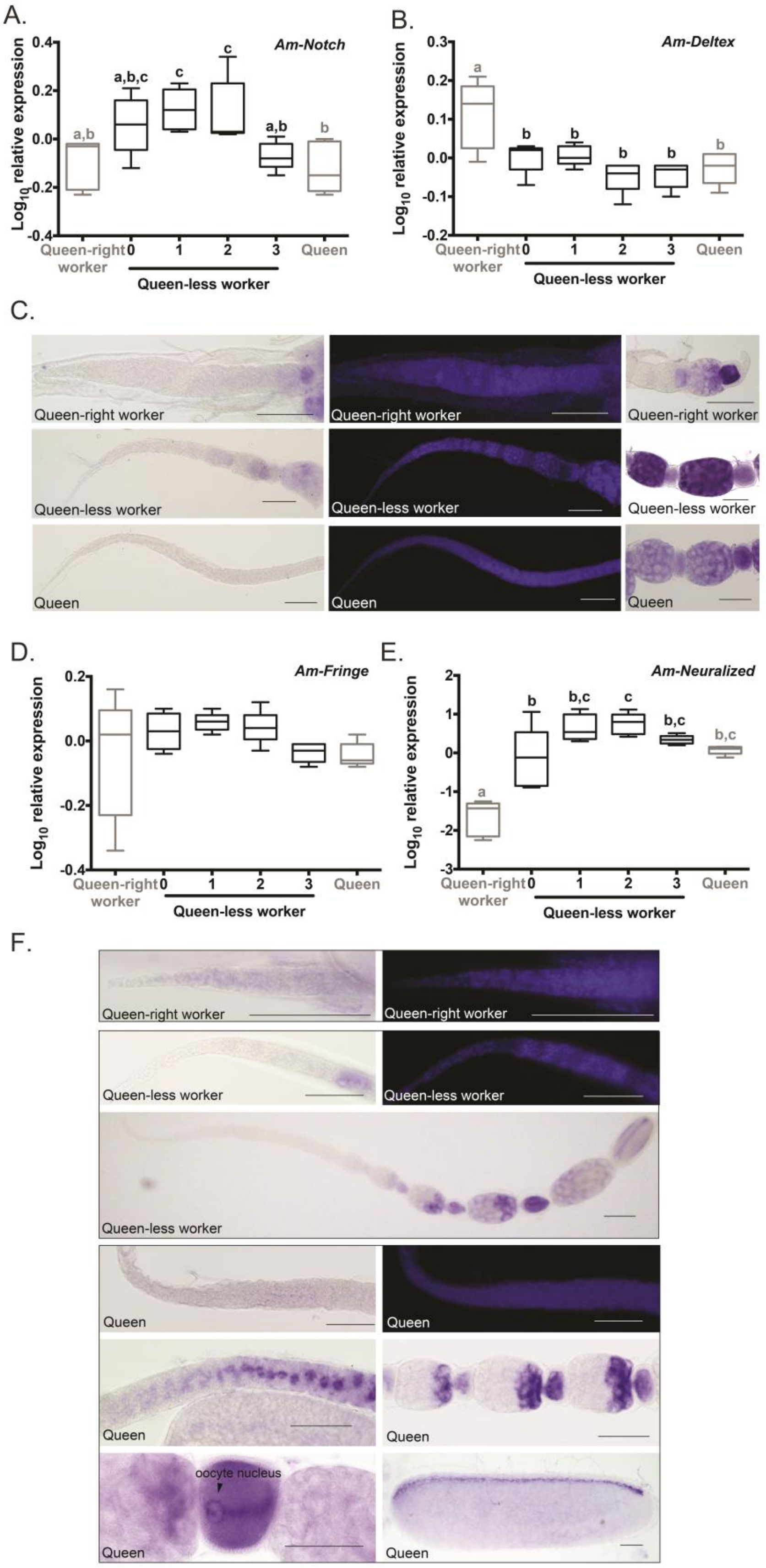
A.
Am-Notch
Log10 relative expression
0.4
0.2
0.0
-0.2
-0.4
a,b
a,b,c
c
c
a,b
b
Queen-right worker
0
1
2
3
Queen
Queen-less worker
B.
Am-Deltex
Log10 relative expression
0.3
0.2
0.1
0.0
-0.1
-0.2
a
b
b
b
b
b
Queen-right worker
0
1
2
3
Queen
Queen-less worker
C.
Queen-right worker
Queen-right worker
Queen-right worker
Queen-less worker
Queen-less worker
Queen-less worker
Queen
Queen
Queen
D.
Am-Fringe
Log10 relative expression
0.2
0.0
-0.2
-0.4
Queen-right worker
0
1
2
3
Queen
Queen-less worker
E.
Am-Neuralized
Log10 relative expression
2
1
0
-1
-2
-3
a
b
b,c
c
b,c
b,c
Queen-right worker
0
1
2
3
Queen
Queen-less worker
F.
Queen-right worker
Queen-right worker
Queen-less worker
Queen-less worker
Queen-less worker
Queen
Queen
Queen
Queen
oocyte nucleus
Queen
Queen

Supplementary Figure 5. RT-qPCR and *in situ* hybridisation of genes related to Notch signalling.

(**A**) *Notch* RNA is transiently induced in queen-less workers with highest expression as the worker ovaries undergo cell differentiation and oocyte specification (score = 0, score = 1). *Notch* RNA levels begin to decline as yolk is detected in the oocytes (score = 2) and decline further as the oocyte matures (score = 3). (**B**) RT-qPCR indicates a subtle, but significant down regulation of *Deltex* in queen-less worker ovaries (compared with queen-right workers). (**C**) *in situ* hybridisation reveals no *Deltex* RNA in the germarium or terminal filament of queen-right workers, queen-less workers or queen ovarioles. *Deltex* RNA is detected in the nurse cells and the developing oocyte in all three ovary types. (**D**) *Fringe* RNA is expressed at equal levels in queen-right worker, queen-less workers (score = 0, 1, 2, 3) and queen ovaries. (**E**) RT-qPCR reveals that *neuralized* RNA is induced very early in the ovary activation process. *Neuralized* RNA peaks in ovaries that are scored as a 2 (as yolk becomes deposited into the oocytes) with neuralized RNA detected at 5-fold higher levels than queen-right controls. Neuralized RNA then declines to queen-like levels in laying-workers (score = 3). (**F**) *neuralized* RNA is detected weakly throughout the ovariole in queen-right worker bees. In queen-less worker ovarioles and queen ovarioles no expression is detected in the terminal filament or germarium. *Neuralized* RNA is detected in the presumptive oocytes as they are specified and RNA accumulates strongly in these oocytes as they enlarge and begin to separate from the associated nurse cells. As the oocyte separates from the nurse cell bundle *neuralized* RNA is only detected in the posterior nurse cells. Late in oogenesis *neuralized* RNA is enriched on the dorsal surface of the oocyte and surrounds the oocyte nucleus. Following *in situ* hybridisation ovaries were counter-stained with DAPI (right panels). Scale bars indicate 100 µm. All RT-qPCR data is the mean of transcript levels (Log_{10}) in five biological samples for each condition. Boxplot whiskers indicate minimum and maximum, the box is defined by 25th percentile, median and 75th percentile. Differences in target gene expression were determined by ANOVA with a Tukey's post-hoc test, statistical differences ($P < 0.05$) are denoted by different letters.

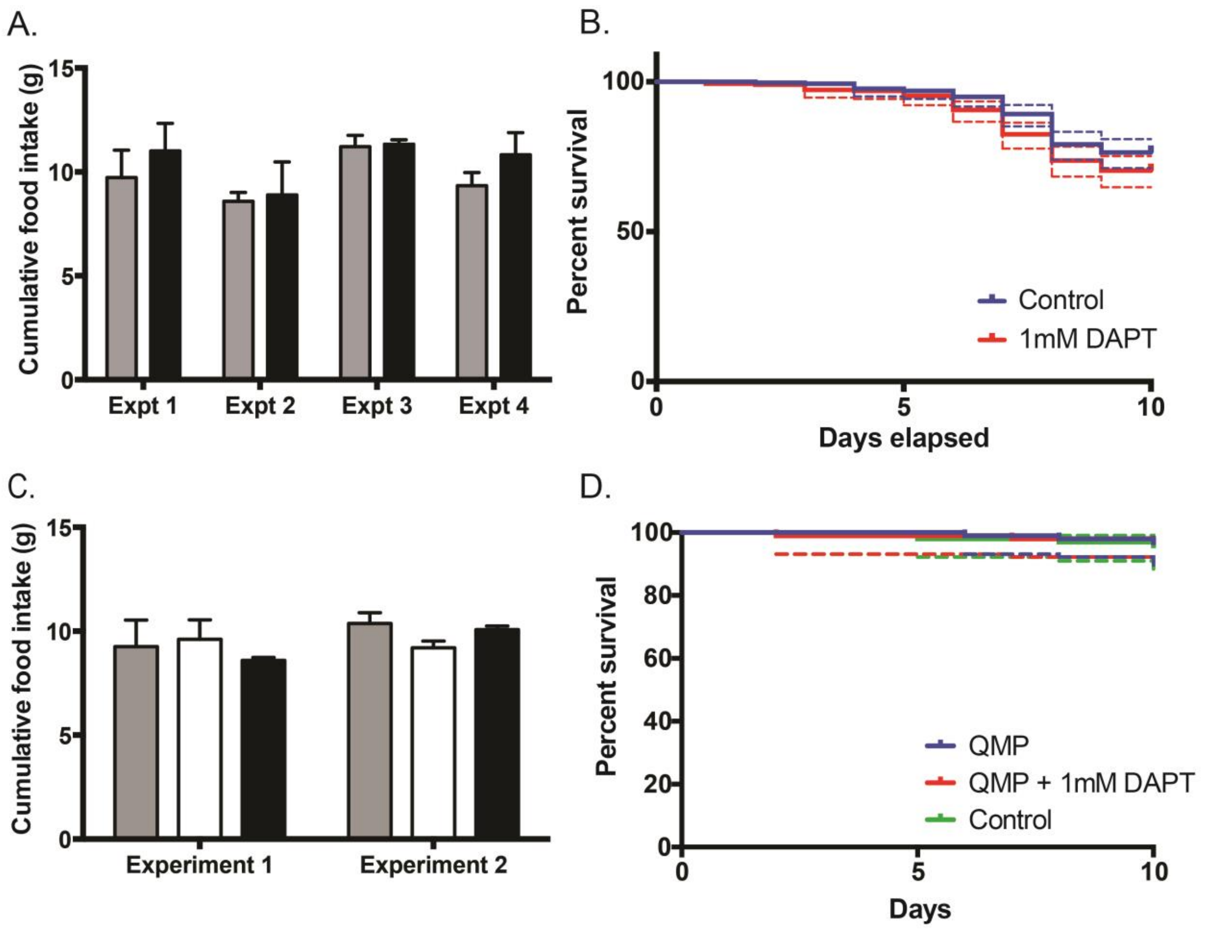

Supplementary Figure 6. Food intake and Kaplan-Meier survival curves for DAPT treated bees.

In the absence of QMP, supplementation of the diet with DAPT had no significant effect on food intake (**A**, grey = control (ethanol), black = DAPT treated (1mM)) or survival (**B**) as compared with the ethanol solvent control. Similarly, in the presence of QMP supplementation of the diet with DAPT had no significant effect on food intake (**C**, grey = control (ethanol), white = QMP control (QMP + ethanol), black = QMP and DAPT treated (1mM)) or survival (**D**) as compared with the ethanol solvent control. Dotted lines on Kaplan-meier survival curves are 95% confidence intervals. Statistical significance for food intake was measured using t-tests with a Holm-Sidak correction for multiple testing and for survival was measured using a log rank test[4].

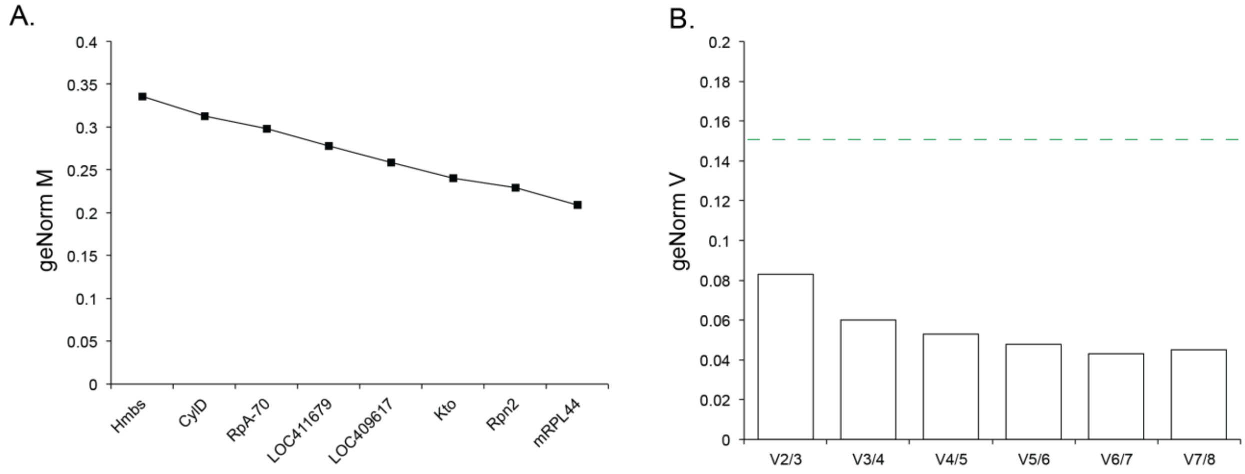

Supplementary Figure 7. Validation for reference genes in the honeybee ovary during ovary activation.

Eight putative reference genes were selected for analysis. (**A**) GeNorm analysis identified Rpn2 and *mRPL44* as the most stable (lowest M-value) across these samples. M-values for all genes were less than 0.5, which is consistent with stable expression in a relatively homogenous cell population[5]. (**B**) Pair-wise variation analysis between the normalization factors to determine the optimal number of reference genes for normalization. GeNorm analysis indicated that expression across these samples was relatively stable and that two reference genes were recommended for normalization of gene expression.

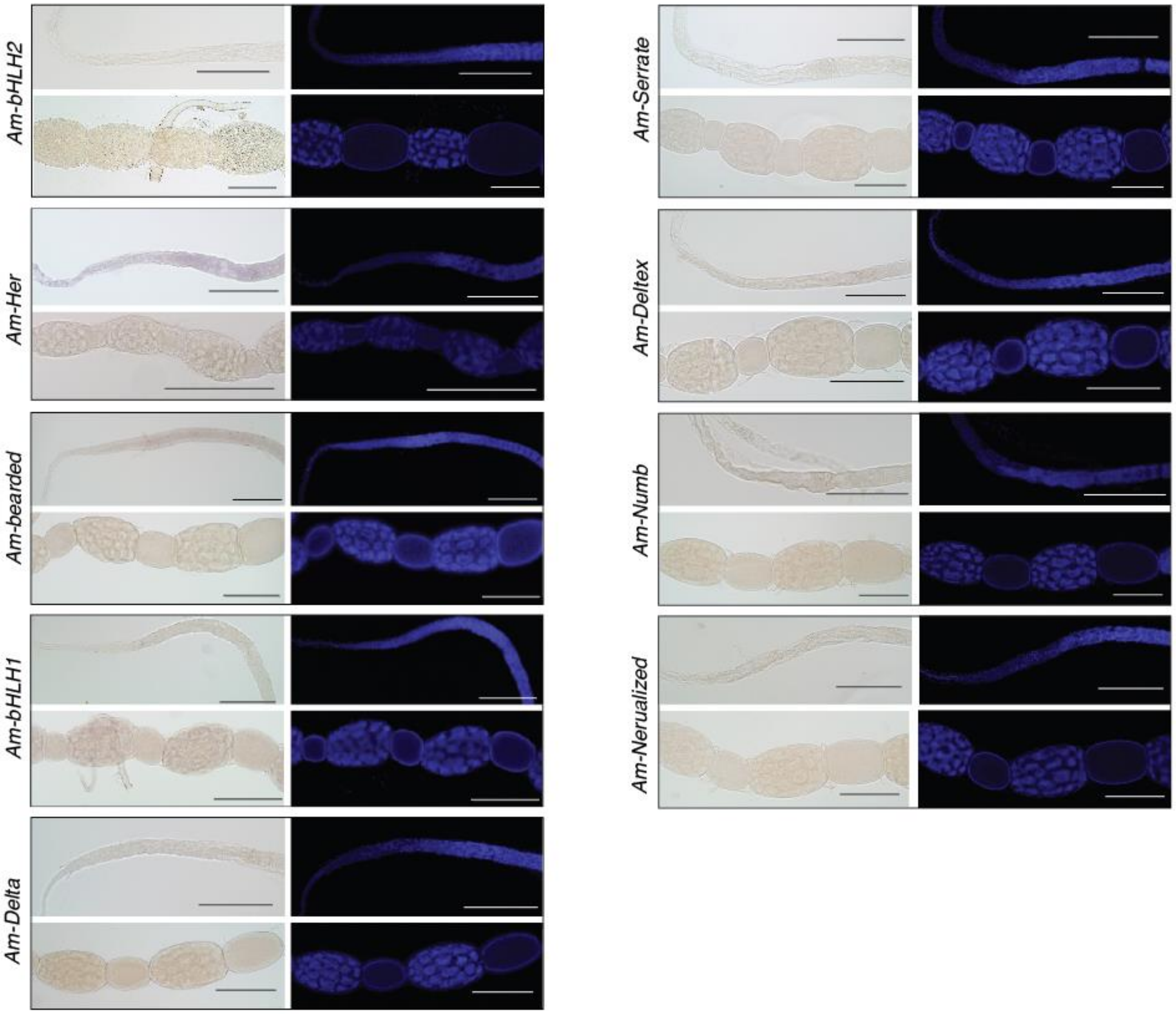

Supplementary Figure 8. Sense probe controls for *in situ* hybridisation experiments.

Queen ovaries were hybridized with equivalent amounts of sense control probes under the same conditions as used for antisense probes (Fig. 2-4, Supplementary Fig. 2, 4, 5). Representative images are shown. Gene names are to the left side of each panel. Within each panel the top left image shows the germarium (and the top right is the corresponding DAPI stained image), the bottom left image shows nurse cells and oocytes between stage 3 and 5 of oogenesis (and the bottom right is the corresponding DAPI image). Following *in situ* hybridisation ovaries were counter-stained with DAPI (right panels). Scale bars indicate 100 µm.

Supplementary Tables

Supplementary Table 1: Gene expression as a predictor of physiological status of the ovary. Red indicates the most reliable predictor in this study, whilst bold font indicates genes that are equivalent or better predictors than a previously published gene, *Anarchy*[6].

Target Gene	% accurate prediction ‘active’ versus ‘inactive’
Notch	40%
Delta	**87%**
Serrate	**93%**
Numb	33%
Fringe	37%
Neuralized	63%
bHLH2	**97%**
bHLH1	33%
Her	**87%**
bearded	50%

Supplementary Table 2: Oligonucleotide sequences used in this study

Target Gene	Honeybee ID*	5' Primer	3' Primer
Oligonucleotide sequences used to detect expression of reference genes by RT-qPCR.			
mRPL44 *(mitochondrial ribosomal protein L44)*	GB53201	GCA TGG TTG CAT ATT GGA ACA	CGC TCC CAC AAG TGC TAG A
Rpn2 *26S proteasome non-ATPase regulatory subunit 1*	GB52526	CGC CTG TAA TGG AAA CTG AAA	ACA CGT TCT TGT TGC TCA CG
Kto: *kohtalo*	GB45373	TCA ACC AGC ACC ACC ATA CC	TGT TGC CTC ATA CGT TGT TGG
LOC411679 *Keap1 (kelch-like ECH-associated protein 1)*	GB41840	TCA AGA GAT TGG GAG TTG GAG	CAT TCA TCA TTT TCA GGA TGG
CylD *cylindromatosis*	GB45155	ACA AAT GCC ACG ATT TGG A	TTT CCG CAA ACA GTA CAT TGT C
Kto *kohtalo*	GB45373	TCA ACC AGC ACC ACC ATA CC	TGT TGC CTC ATA CGT TGT TGG
Hmbs *(lethal (3) 02640, porphobilinogen deaminase)*	GB52351	CCT GAT GAT GAT GGA GAA CCT C	CGA GTT GTT CAG CAC CCA AA
*LOC409617**ArfGAP3 (ADP-ribosylation factor GTPase activating protein 3)*	GB48600	CCA AAG CAA CAC AAG CAG AA	TTA TTG TTC TCA TAT CAC CAA GTG C
RpA-70 *(Replication Protein A 70)*	GB44421	TTC AGG TAG AGA ATT AAG GAA ACG A	TTC AGC TTG TGA TCC CCA TA
Oligonucleotide sequences used to detect expression of target genes by RT-qPCR.			
bearded	GB43776	CAG TTG TTC CCG TTG TTC CT	GCT CAG GGT CGT CCA GTA GA
bHLH1	GB43778	GCA GAT GCA CGA ACA GAT CA	TTG CGT TCC AAC ATA GGT TTC

bHLH2	GB43790	GGG AAG CGG GAT CAA GAT A	AGT CTG GGC GAG GAG ATG TA
Delta	GB45005	AGG GCG ACT ACT GCA ATA CC	AGT ACG CTC CCT TCC AAC C
Deltex	GB51755	CCT GGA ACT ATG AAT TGG ACA AG	GGA TTA GGA TGT TCT CTT CCT TGA
Fringe	GB44913	GCG TAA AGA CCA CCA AGC AT	TGG AAA TAC GGA TCG TCT CG
Her	GB43788	ACC ACC ACC GTA GCA TCA TC	ACT TTG GGG AGG CGT GTA A
Neuralized	GB55094	CGC CGT TAC CCT TCC ACT	ATT CCG TGT CGG TCC TTG T
Notch	GB49149	TGA TAC CAA ACG GTC CTC TCA	GCT TCT TCG ACT TCG CTT GT
Numb	GB48034	TAG AGA CGG CAC GAC CAG AC	CAG CAA AAG CAC ATC CCA CT
Serrate	GB48639	CCA ATT CGA CGT GGA TGA GT	CAG TTC TTC CCC GTG AAA CC
Oligonucleotide sequences used to clone genes for in situ hybridisation probes			
bearded	GB43776	CAC TAT GAG CCA CGT GTT CG	ATC ACT GTC GGT CAT GCT CA
bHLH1	GB43778	CCT TGG GAA CCA AAT TGA TG	TCG ACG GGA GAA AAA TAT CG
bHLH2	GB43790	ACG AAG CCA TTG TTG GAA AG	GCC GCC ACA TAG GAT CAA
Deltex	GB51755	TTT GGA AAA CCA GGC AGT G	TGT TGT TGT GTG GGA ATG G
Her	GB43788	CAC CGC ACA CCA GCT ACA C	TCA CCA AGG CCT CCA AAC
Neuralized	GB55094	GAC CCG ATC AAC CTC AGA AA	CCA CAA TTG AAG GCT TTG GT
Numb	GB48034	AGT GTG CGA GGA AGC TCT AAA	GGA TGG GAC TGA GAT TGC TG
Serrate	GB48639	GAT TCG GGA TCA ACT GGT TC	TCT GCT CTT TCA TAG GGT TCG

* gene identifiers are from BeeBase genome assembly v4.5 official gene set version 3.2

Supplementary Notes

Supplementary Note 1

Determination of appropriate reference genes for qRT-PCR analysis. One of the critical steps for obtaining reliable quantification of gene expression by qRT-PCR is identification and use of reference genes that are stable across cell types and treatment conditions. To identify suitable reference genes for use in the ovaries of queen, worker and queen-less worker bees we examined the expression stability of eight reference genes (for primer sequences refer to Supplementary Table 2) across three samples from each of the treatment conditions (queen-right worker, queen-less worker (score = 0, score = 1, score = 2, score = 3) and queen ovaries) used in this study.

PCR efficiencies for each of the possible reference genes were determined using a 10 fold dilution series of ovary cDNA. The PCR efficiencies ranged from 95% to 105% with R^2 values > 0.98. Analysis of the melting profiles showed no evidence of primer dimer, or amplification from genomic DNA (data not shown) and specificity of the amplification was confirmed by direct sequencing of the PCR product.

Raw expression data was analyzed using GeNormPlus. All eight of the candidate reference genes had high expression stability (M-values < 0.4, Supplementary Fig. 7A). GeNorm indicated that for the experimental conditions tested (encompassing the full range of ovary activation illustrated in Supplementary Fig. 1B) that the geometric mean of two reference genes was optimal for normalization of the qRT-PCR data (Supplementary Fig. 7B). *Am-Rpn2* (26S proteasome non-ATPase regulatory subunit 1) and *Am-mRPL44* (Mitochondrial ribosomal protein L44) were chosen for use in this study has they have the highest stability values (lowest M-values, Supplementary Fig. 7B).

Genes of the E(spl)-C are expressed in overlapping domains of the honeybee ovary. The four genes of the E(spl)-C (*bHLH1*, *Her*, *bearded*, and *bHLH1*) are orthologous to the *Drosophila* E(spl)-C genes[7] which have well-defined roles in neurogenesis, are expressed in a Notch responsive manner and depend on Su(H) for initiation and maintenance of gene expression[8-11]. Expression of these genes during embryonic development is consistent with these genes also being regulated by Notch signalling in honeybees[7].

Expression of these genes in the honeybee ovary varies with reproductive status (Fig. 2). RNA for all four of these genes is detected in the germarium of queen-right but not queen-less or queen ovarioles (Fig. 2).

In the upper germarium, as the presumptive oocyte is specified from the cystocyte cluster, *bearded* RNA is detected in the presumptive oocytes (Supplementary Fig. 2C). *Bearded* RNA accumulates in the presumptive oocyte before it is detected in the associated nurse cells. As oogenesis proceeds *bearded* RNA becomes detectable in the nurse cells and continues to accumulate in the developing oocyte until it matures.

Genes of the E(spl)-C genes are, however, expressed more extensively, particularly in the queen ovary and queen-less worker ovary suggesting that Notch signalling also has other roles oogenesis. RNA for all four of these genes is detected in the nurse cell clusters, and three of these RNAs (*bHLH2*, *bearded* and to a lesser degree, *Her*) are maternally provided to the developing oocyte (Supplementary Fig. 2A,B,C). In contrast *bHLH1* RNA is detected relatively weakly in the oocytes later in oogenesis (from stage 5 onwards) (Supplementary Fig. 2D). Maternal provision of these RNAs to the developing oocyte may suggest a possible role for these genes in regulating oocyte maturation or early developmental processes.

Notch signalling differentiates follicle cell populations in the honeybee queen ovary. In *Drosophila* Notch signalling has multiple roles in oogenesis; in addition to the requirement for Notch signalling in germ cell proliferation and maintenance, Notch signalling is also required for the proliferation, migration and differentiation of the follicle cells. Follicle cells are a somatic cell lineage that encapsulate the developing oocyte and are important for establishing the anterior-posterior and dorso-ventral axes of the oocyte[12]. Early in oogenesis in *Drosophila*, Delta signalling from the germ line activates the Notch receptor in neighbouring follicle cells and

patterns these cells in conjunction with JAK/STAT signalling[13,14]. These patterning events act to differentiate these cells into three distinct cell populations; main body follicle cells, polar follicle cells and stalk cells. Patterning of the follicle cells into these populations is required for achieving the correct morphology of individual egg chambers and axial patterning[15].

It has previously been reported that Notch signalling may play a similar role in regulating follicle cell migration and specification in the honeybee[16]. In the queen honeybee, *Delta* RNA is detected in the presumptive oocytes soon after they are specified, and is readily detectable by stage 1 of oogenesis (Fig. 3). *Delta* RNA continues to accumulate in the oocyte throughout oogenesis persisting until the oocyte is mature[16]. Using immunohistochemistry for the Notch intracellular domain we were able to show that the Notch receptor is present on all of the nurse cell membranes and on the follicle cell membranes (Supplementary Fig. 4A,B). By mid-late oogenesis (stage 3 to stage 6) intracellular accumulation of the Notch receptor is observed in the anterior and posterior follicle cells (arrows in Supplementary Fig. 4B). These intracellular foci of Notch protein do not co-localize with follicle cell nuclei or F-actin (Supplementary Fig. 4C,D).

To determine whether the Notch intracellular domain is colocalized with recycling endosomes we used immunohistochemistry with the Notch intracellular domain and *Drosophila* Rab11 as a marker of recycling endosomes. The Notch intracellular domain does not colocalize with recycling endosomes (Supplementary Fig. 4E,F), raising the possibility that the Notch intracellular domain may be sequestered intracellularly associated with degradative endosomes. This sequestration may ensure that these anterior and posterior follicle cells are refractory to Notch signalling, and likely acts to differentiate these follicle cell populations. Consistent with this hypothesis, RNA for the E(spl)-C genes is detected at the apical surface of all of the main body follicle cells (Supplementary Fig. 4G, H), suggesting that these cells are receiving the Notch signal from either Delta or Serrate present in the oocyte. However, RNA for the E(spl)-C genes is not detected in the anterior and posterior follicle cells (Supplementary Fig. 4G,H, and confocal reflectance in I, J), confirming that these cells are not receiving a Notch signal.

While Notch signalling has a role in differentiating follicle cell populations in *Drosophila* and the honeybee, the details of the way that Notch signalling achieves this differs. In *Drosophila*, Delta signalling from the germ-line stimulates Notch receptor activation in the polar follicle cells, whereas it is the anterior and posterior follicle cells that are refractory to Notch

signalling in the honeybee. These data imply that over evolutionary time the regulation of this pathway has altered but the specification of sub-populations of follicle cells is still dependent on Notch signalling from the germline.

Expression of the gene encoding the Notch receptor. Differences observed in Notch signalling between queen-less and queen-right worker ovaries (Fig. 2) could be due to differential expression of the *Notch* mRNA and protein. RT-qPCR demonstrates that mRNA levels for the *Notch* receptor are dynamic during the ovary activation process with a modest increase (1.7 fold) in *Notch* mRNA levels seen prior to yolk being deposited into the oocyte (score = 2). Notch RNA levels decline as queen-less worker ovaries become fully mature and produce oocytes (Supplementary Fig. 5A). This dynamic expression, where queen-right workers have relatively low levels of *Notch* mRNA does not explain the high levels of Notch signalling we observe in queen-right worker ovaries (Fig. 2).

Expression of *Deltex*. Deltex, a cytoplasmic ring-domain ubiquitin ligase, has been implicated in ligand-independent activation of Notch signalling[17]. Cleavage of the Notch receptor and release of the Notch intracellular domain is dependent on sequestration of the Notch protein in the lysosome limiting membrane[18]. This non-canonical activation of Notch may have roles in multiple developmental contexts; including hematopoiesis and neurogenesis in *Drosophila*[17]. To determine if Deltex may be regulating the degradation or activation of the Notch receptor in the germarium we first examined expression of *Deltex* RNA by RT-qPCR and then examined which cells of the ovary were expressing *Deltex* RNA by *in situ* hybridisation. RT-qPCR shows that *Deltex* RNA is subtly but consistently down-regulated in queen-less worker ovaries as compared to queen-right workers (Supplementary Fig. 5B). *In situ* hybridisation revealed very similar expression patterns between queen-right, queen-less and queen worker ovaries (Supplementary Fig. 5C). *Deltex* RNA is expressed by nurse cells and is deposited into the developing oocyte, but is not detected in the germarium or terminal filament. This is consistent with Deltex having a role late in oogenesis, but not affecting Notch signalling in the germarium.

Expression of *Fringe.* The activity of the Notch signalling pathway is modulated by a large number of proteins that can influence signalling both positively and negatively, these include

Fringe, which glycosylates the Notch receptor increasing the affinity of the receptor for the Delta ligand[19], and possibly providing ligand specificity. *Am-Fringe* is not differentially expressed between queen-right worker bees and queen-less worker bees (Supplementary Fig. 5D), indicating that *Fringe* does not have a role the differential regulation of Notch signalling observed in queen-less and queen-right worker ovaries

Expression of *Neuralized*. Neuralized is a ubiquitin ligase that is responsible for the endocytosis and ubiquitination of Delta[20]. The endocytosis and ubiquitination of both Delta and Serrate potentiates Notch signalling activity through an unknown mechanism[21-23].

RT-qPCR shows that expression of *Am-neuralized* is induced in queen-less worker bees very early in the ovary activation process, even before the ovaries are morphologically distinguishable from queen-right worker ovaries (3 fold induction in queen-less worker ovaries (score = 0) compared to queen-right workers). Expression peaks at levels 5-fold higher than queen-right workers in queen-less workers score = 2 (just as yolk begins to be deposited into the oocyte) (Supplementary Fig. 5E). *In situ* hybridisation reveals that *neuralized* RNA is detected very weakly in the germarium of queen-less and queen bees but accumulates strongly in the newly specified oocytes (Supplementary Fig. 5F). Mid-way through oogenesis *neuralized* RNA becomes enriched at the dorsal surface of the oocyte and this enrichment persists as the oocyte matures (Supplementary Fig. 5F). This expression pattern is similar to that seen for *tailless* in honeybees which has a role in patterning early honeybee embryos[24]. During oogenesis *tailless* RNA is localised to the dorsal surface enriching in the posterior as the oocyte matures[42]. During early embryogenesis maternal *tailless* RNA moves to the posterior of the oocyte[42] and RNA interference studies have shown that it has a role in posterior patterning[42]. The similar localization of *neuralized* RNA to the dorsal surface of the developing oocyte may indicate that it has novel functions in axis formation or embryonic patterning. A previous study, however, demonstrated that Notch signalling does not have a role in segmentation in the honeybee[25]. In-depth analysis, such as parental RNAi, of *neuralized* function is required to determine if neuralized has a role in early development in the honeybee.

Gene expression as a predictor of worker ovary state. A recent study demonstrated, using Decision Tree Recursive Partitioning[26], that expression of a single gene, *Anarchy,* in the

honeybee ovary is capable of classifying the physiological state of the ovary (as either active or inactive) 88.2% of the time[6]. It is argued that the relationship between physiological state of the ovary and *Anarchy* expression, together with other features of *Anarchy* expression, such as the sensitivity of *Anarchy* expression in worker ovaries to the presence of the queen, that *Anarchy* a gene underlying altruism[6,27].

To compare the genes measured in this study with *Anarchy* we also performed Decision Tree Recursive Partitioning as detailed for *Anarchy*[6] (Supplementary Table 1). In contrast with *Anarchy,* which only predicts physiological state of the ovary 88.2% of the time [6], we found that expression of the Notch responsive gene *bHLH2* predicts physiological state of the ovary 97% of the time. Expression of another Notch responsive gene, *Her*, predicts the physiological state of the ovary 87% of the time. Expression of the ligands for the Notch receptor, *Delta* and *Serrate*, predict the physiological state of the ovary 93% and 87% of the time, respectively.

Genes involved in Notch signalling also meet the testable-criteria outlined by Thompson[27] for genes underlying altruism; the expression of many of these genes associated with Notch signalling are responsive to the social environment (including *bHLH2, Her, bearded, Delta, Serrate, Numb, Notch, Deltex* and *Neuralized*). With the exception of *Notch* and *Numb* these genes are also differentially expressed in the ovaries of adult worker bees and queen bees; this reflects a theoretical constraint of altruistic genes linked to QMP exposure as genes must only be responsive to QMP in worker ovaries to prevent queens sterilizing themselves with their own pheromone[6].

In addition to meeting the testable criteria for genes underlying altruism[27] we demonstrate, using functional manipulation, that inhibition of Notch signalling can overcome the repressive effect of QMP on ovary activity and that Notch signalling acts on the earliest stages of oogenesis, in the germarium, the region of the ovary that has been shown to differ morphologically between queen-right and queen-less worker bees[1]. We therefore conclude that Notch signalling is the proximate mechanism maintaining reproductive sterility in the worker honeybee and this sterility is crucial for conflict resolution over production of males[28] and social harmony[29,30].

Supplementary References

1 Tanaka, E. D. & Hartfelder, K. The initial stages of oogenesis and their relation to differential fertility in the honey bee (*Apis mellifera*) castes. *Arthropod structure & development* **33**, 431-442 (2004).

2 Hess, G. in *Ein Beitrag zur Frage der Regulationen im Bienenstaat, Beih. Schweiz. Bienen Ztg. 2.* 33-111 (1942).

3 Fehon, R. G. *et al.* Molecular interactions between the protein products of the neurogenic loci *Notch* and *Delta*, two EGF-homologous genes in *Drosophila*. *Cell* **61**, 523-534 (1990).

4 Rich, J. T. *et al.* A practical guide to understanding Kaplan-Meier curves. *Otolaryngology--head and neck surgery : official journal of American Academy of Otolaryngology-Head and Neck Surgery* **143**, 331-336 (2010).

5 Hellemans, J., Mortier, G., De Paepe, A., Speleman, F. & Vandesompele, J. qBase relative quantification framework and software for management and automated analysis of real-time quantitative PCR data. *Genome biology* **8**, R19 (2007).

6 Ronai, I. *et al.* Anarchy Is a Molecular Signature of Worker Sterility in the Honey Bee. *Molecular biology and evolution* (2015).

7 Duncan, E. J. & Dearden, P. K. Evolution of a genomic regulatory domain: the role of gene co-option and gene duplication in the Enhancer of split complex. *Genome Res* **20**, 917-928 (2010).

8 Wurmbach, E., Wech, I. & Preiss, A. The Enhancer of split complex of *Drosophila melanogaster* harbors three classes of Notch responsive genes. *Mechanisms of development* **80**, 171-180 (1999).

9 Lai, E. C., Bodner, R. & Posakony, J. W. The enhancer of split complex of *Drosophila* includes four Notch-regulated members of the bearded gene family. *Development* **127**, 3441-3455 (2000).

10 Bailey, A. M. & Posakony, J. W. Suppressor of hairless directly activates transcription of enhancer of split complex genes in response to Notch receptor activity. *Genes & development* **9**, 2609-2622 (1995).

11 Lecourtois, M. & Schweisguth, F. The neurogenic suppressor of hairless DNA-binding protein mediates the transcriptional activation of the enhancer of split complex genes triggered by Notch signaling. *Genes & development* **9**, 2598-2608 (1995).

12 Roth, S. & Lynch, J. A. Symmetry breaking during *Drosophila* oogenesis. *Cold Spring Harbor perspectives in biology* **1**, a001891 (2009).

13 McGregor, J. R., Xi, R. & Harrison, D. A. JAK signaling is somatically required for follicle cell differentiation in *Drosophila*. *Development* **129**, 705-717 (2002).

14 Roth, S. *Drosophila* oogenesis: coordinating germ line and soma. *Current biology : CB* **11**, R779-781 (2001).

15 Assa-Kunik, E., Torres, I. L., Schejter, E. D., Johnston, D. S. & Shilo, B. Z. *Drosophila* follicle cells are patterned by multiple levels of Notch signaling and antagonism between the Notch and JAK/STAT pathways. *Development* **134**, 1161-1169 (2007).

16 Wilson, M. J., Abbott, H. & Dearden, P. K. The evolution of oocyte patterning in insects: multiple cell-signaling pathways are active during honeybee oogenesis and are likely to play a role in axis patterning. *Evolution & development* **13**, 127-137 (2011).

17 Baron, M. Endocytic routes to Notch activation. *Seminars in cell & developmental biology* (2012).

18 Fortini, M. E. & Bilder, D. Endocytic regulation of Notch signaling. *Current opinion in genetics & development* **19**, 323-328 (2009).

19 Bruckner, K., Perez, L., Clausen, H. & Cohen, S. Glycosyltransferase activity of Fringe modulates Notch-Delta interactions. *Nature* **406**, 411-415 (2000).

20 Lai, E. C., Deblandre, G. A., Kintner, C. & Rubin, G. M. *Drosophila* neuralized is a ubiquitin ligase that promotes the internalization and degradation of delta. *Developmental cell* **1**, 783-794 (2001).

21 Le Borgne, R. Regulation of Notch signalling by endocytosis and endosomal sorting. *Current opinion in cell biology* **18**, 213-222 (2006).

22 Le Borgne, R., Bardin, A. & Schweisguth, F. The roles of receptor and ligand endocytosis in regulating Notch signaling. *Development* **132**, 1751-1762 (2005).

23 Weinmaster, G. & Fischer, J. A. Notch ligand ubiquitylation: what is it good for? *Developmental cell* **21**, 134-144 (2011).

24 Wilson, M. J. & Dearden, P. K. Tailless patterning functions are conserved in the honeybee even in the absence of Torso signaling. *Developmental biology* **335**, 276-287 (2009).

25 Wilson, M. J., McKelvey, B. H., van der Heide, S. & **Dearden, P. K.** Notch signaling does not regulate segmentation in the honeybee, *Apis mellifera*. *Development genes and evolution* **220**, 179-190 (2010).

26 Quinlan, J. R. *C4.5 : programs for machine learning*. (Morgan Kaufmann, 1993).

27 Thompson, G. J., Hurd, P. L. & Crespi, B. J. Genes underlying altruism. *Biology letters* **9**, 20130395 (2013).

28 Ratnieks, F. L., Foster, K. R. & Wenseleers, T. Conflict resolution in insect societies. *Annual review of entomology* **51**, 581-608 (2006).

29 Khila, A. & Abouheif, E. Reproductive constraint is a developmental mechanism that maintains social harmony in advanced ant societies. *Proceedings of the National Academy of Sciences of the United States of America* **105**, 17884-17889 (2008).

30 Khila, A. & Abouheif, E. Evaluating the role of reproductive constraints in ant social evolution. *Philosophical transactions of the Royal Society of London. Series B, Biological sciences* **365**, 617-630 (2010).
